# Identification of a Common Epitope between Enterovirus 71 and Human MED25 Proteins Which May Explain Virus-Associated Neurological Disease

**DOI:** 10.3390/v7041558

**Published:** 2015-03-27

**Authors:** Peihu Fan, Xiaojun Li, Shiyang Sun, Weiheng Su, Dong An, Feng Gao, Wei Kong, Chunlai Jiang

**Affiliations:** 1 School of Life Sciences, Jilin University, Changchun 130012, China; E-Mails: fph121@163.com (P.F.); lixiaojun3345@163.com (X.L.); sunsy121@163.com (S.S.); suweiheng@163.com (W.S.); andonginlab@sina.cn (D.A.); feng0215@gmail.com (F.G.); 2 National Engineering Laboratory for AIDS Vaccine, Jilin University, Changchun 130012, China; 3 Key Laboratory for Molecular Enzymology and Engineering, Jilin University, Changchun 130012, China

**Keywords:** enterovirus 71, common antigen, MED25, neurological disease

## Abstract

Enterovirus 71 (EV71) is a major causative pathogen of hand, foot and mouth disease with especially severe neurologic complications, which mainly account for fatalities from this disease. To date, the pathogenesis of EV71 in the central neurons system has remained unclear. Cytokine-mediated immunopathogenesis and nervous tissue damage by virus proliferation are two widely speculated causes of the neurological disease. To further study the pathogenesis, we identified a common epitope (co-epitope) between EV71 VP1 and human mediator complex subunit 25 (MED25) highly expressed in brain stem. A monoclonal antibody (2H2) against the co-epitope was prepared, and its interaction with MED25 was examined by ELISA, immunofluorescence assay and Western blot *in vitro* and by live small animal imaging *in vivo*. Additionally, 2H2 could bind to both VP1 and MED25 with the affinity constant (Kd) of 10^−7^ M as determined by the ForteBio Octet System. Intravenously injected 2H2 was distributed in brain stem of mice after seven days of EV71 infection. Interestingly, 2H2-like antibodies were detected in the serum of EV71-infected patients. These findings suggest that EV71 infection induces the production of antibodies that can bind to autoantigens expressed in nervous tissue and maybe further trigger autoimmune reactions resulting in neurological disease.

## 1. Introduction

Enterovirus 71 (EV71) is the predominant pathogen of hand, foot and mouth disease (HFMD), which is mainly characterized by low-grade fever for three to four days and vesicles on the hands, feet and oropharyngeal mucosa of infected children of <5 years of age. Occasionally, severe neurological disease such as encephalitis, aseptic meningitis and pulmonary edema caused by EV71 infection may occur and lead to death [1,2,3]. EV71 is a member of the *Enterovirus* genus in the *Picornaviridae* family, which consists of a positive-sense, single-stranded RNA and a non-enveloped icosahedral capsid containing four structural proteins: VP1, VP2, VP3 and VP4. Among these proteins, VP1 is widely accepted to play critical roles in virus entry and uncoating [4] and contains many critical neutralization sites [5,6,7]. Since the first identification of EV71 in the United States in 1969 [8], outbreaks of infection with this virus have occurred worldwide, especially in countries of Southeast Asia in recent years, including Mainland China, Taiwan, Malaysia, Singapore and Brunei [9,10,11,12,13]. Since the outbreak of EV71 infection in Fuyang of Anhui Province in 2008, infections of this virus have spread dramatically throughout the People’s Republic of China (PRC). More importantly, the morbidity and mortality (especially severe cases of neurological disease) of HFMD have increased over time [11]. This trend suggests that the morbidity of severe neurological complications is an increasing threat to public health. Therefore, clearly understanding the pathogenesis of EV71 in the nervous system is important in efforts to control the neurological disease caused by EV71 infection.

Many studies have reported on the potential neurological pathogenesis of EV71. The emergence of strains with enhanced virulence was considered to be the main reason for frequent outbreaks and more severe clinical manifestations [14]. EV71 strains from encephalitis patients were identified as having highly enhanced neurotropism as well as greater cytotoxicity [15,16,17]. Therefore, when the EV71 infection reaches neurological tissues, the replicating virus will cause lesions that lead to the neurological symptoms. However, the cell types initially infected when the enterovirus invades the body, the specific route of migration to the central nervous system, as well as the determinant of the neurotoxicity of the virus have not been determined [18,19]. An additional perplexing aspect of this condition is that the virus is generally not detected in the cerebrospinal fluid or blood of patients, even those with severe neurological disease [20,21].

The other potential neurological pathogenesis of EV71 is autoimmune-mediated neural lesions induced by host immune response to virus infection. Both innate and adaptive immune responses are important to protect the host from infection. During the period when the virus triggers the host innate immune system, which in turn activates the adaptive immune system, many types of inflammatory factors, cytokines and chemokines are generated. These factors have been demonstrated to play key roles in the pathophysiology of viral infection [22]. The surge of cytokine production and the persistence of hypercytokinemia (namely cytokine storm) may lead to multiple organ disorder [23]. Some reports have indicated that interleukin 6 (IL-6), tumor necrosis factor α (TNF-α) and IL-1β contribute to EV71-induced brain stem encephalitis (BE) and pulmonary edema (PE) [24,25], and highly increased levels of IL-10, IL-13 and interferon γ (IFN-γ) have been detected in plasma of EV71-infected patients with PE [26]. The IL-6 level in plasma was shown to be significantly elevated in patients with autonomic nervous system (ANS) dysregulation [27]. IL-10, IL-13 and IFN-γ are also associated with the neuropathic disease as reviewed previously [28]. All of these findings suggest that the immune response is associated with the neurological complications of EV71 infection, although the specific mechanism of this pathogenesis remains unclear.

A recent report indicated that EV71-induced antibodies could cross-react with brain tissue in mice and human [29]. This finding suggests the existence of a common antigen between EV71 and brain tissue, which can induce the generation of antibodies that react with the virus and host antigen simultaneously, possibly leading to autoimmune-mediated neural lesions. The aim of the present study was to identify the common antigen and investigate the cross-reactivity of EV71-induced antibodies with human brain tissue, which may explain ultimate pathogenesis of the neurological disease or may provide a theoretical foundation for further study of EV71 pathogenesis. We identified a common epitope (PPGAPKP) between the EV71 VP1 protein and the human mediator complex (required for gene transcription by RNA polymerase II, which contains 30 subunits in mammals) subunit 25 (MED25 or ARC92) with a high expressing level in brain stem [30]. This evolutionarily conserved protein complex control and regulate transcription by recruiting of RNA polymerase ΙΙ to promoters [31,32]. A monoclonal antibody (mAb, designated 2H2) to the common epitope was prepared, which showed high affinity to MED25 *in vitro*. Intravenously injected 2H2 was distributed in brain stem of mice after seven days of EV71 infection *in vivo*. Interestingly, EV71-infected patient sera were found to contain high levels of 2H2-like antibodies. All of these results suggest that EV71 infection causes the host immune system to generate a high level of self-reactive antibodies, which may result in autoimmune lesions of nervous tissue and consequent neurological disease. By identifying a common epitope between human and EV71 antigens for the first time, this study provides strong evidence to support that the immune response is related to the neurological complications of viral infection. These studies also shed new light on the pathogenesis of EV71-related neurological disease and cautions researchers to avoid this common epitope in future vaccine design.

## 2. Materials and Methods

### 2.1. Ethics Statement

All subjects gave their informed consent for inclusion before they participated in the study. The study was conducted in accordance with the Declaration of Helsinki, and the protocol was approved by the Ethics Committee of Jilin University. The animal trials in this study were carried out in accordance with the Regulations for the Administration of Affairs Concerning Experimental Animals approved by the State Council of People’s Republic of China (11-14-1988). All animal procedures were approved by the Institutional Animal Care and Use Committee (IACUC) of Jilin University (Permit Number: SCXK 2013-0001).

### 2.2. Peptide Scanning for Common Epitope

Peptide scanning for a common epitope between EV71 and human proteins was carried out through a total of 10 BLASTP searches of the human proteome in Genbank using overlapping sets of EV71 protein fragments. In the first round, the EV71 proteome was divided into 10-amino-acid (about as long as an epitope) fragments from the first amino acid and then used to perform the BLASTP searches. The subsequent nine rounds of BLAST searches were conducted similarly with sets of 10-amino-acid fragments of the EV71 proteome cut starting at the 2nd to 11th amino acids and so on. The longest matched epitope was selected for the following experiments.

### 2.3. Determination of Location of Common Epitope

The crystal structure of human EV71 was downloaded from the PDB Protein Data Bank (PDB file ID: 3VBS) and rendered using VMD software (version 1.9.2, downloaded from Theoretical and Computational Biophysics Group). The software settings as recommended by the manual were used to generate the images.

### 2.4. Preparation of 2H2 mAb and EV71 Neutralization Assay

The 2H2 mAb was prepared by hybridoma technology according to a previously reported method [33]. The neutralization assay was carried out using an EV71 pseudovirus-based assay established by our team [34].

### 2.5. Expression of MED25 in 293T Cells

293T cells were obtained from the American Type Culture Collection (ATCC) and maintained in Dulbecco’s modified Eagle medium (DMEM) (Sigma, Saint Louis, MO, USA) supplemented with 10% Fetal bovine serum (FBS) (10% FBS-DMEM). Cells were maintained at 37 °C with 5% CO_2_. The plasmid for expression of human MED25 (GenBank accession number: NM_030973.3) in mammalian cells was constructed by inserting the cDNA into the pcDNA3.1(-) vector (Invitrogen, Waltham, MA, USA). Expression was conducted by transfection into 293T cells using Lipofectamine 2000 (Invitrogen) according to the product manual.

### 2.6. Immunizations

Rabbits were housed in the Experimental Animal Center Laboratory of Jilin University. For the first immunization, recombinant truncated MED25 (truncated protein which retained the common epitope at the C-terminus was expressed by transformation of *E. coli*, see Supplementary File Figure S1) and VLP were, respectively, mixed with Freund’s complete adjuvant at the ratio of 1:1 and then emulsified. The emulsified mixture was injected subcutaneously into rabbits at multiple sites in the back (1 mg protein/rabbit). Freund’s incomplete adjuvant was used for the second immunization at 14 days after the first one. The third immunization was performed in the same manner as the second one. The fourth immunization as the boost was performed 7 days after the third immunization. Serum samples were collected at each immunization time.

### 2.7. Western Blot

The samples (cells or protein solution) were mixed with an equal volume of 2× sample buffer (0.5 M Tris-HCl pH 6.8, 25%; 0.27 M 2-mercaptoethanol; SDS, 0.14 M; bromophenol blue, 0.003 M; glycerol, 20%; H_2_O, 50%) and then boiled for 15 min, followed by centrifugation at 900× *g*, for 30 min. The supernatant of each sample (5 μL, about 2 μg protein) was loaded on the gel and electrophoresed for 1.5 h at 120 V in a Mini-PROTEAN Tetra cell (BIO-RAD, Hercules, CA, USA). The protein bands were transferred from the gel onto a nitrocellulose membrane (BIO-RAD) using a Trans-Blot SD Semi-Dry Electrophoretic Transfer Cell (BIO-RAD) with the voltage setting at 15 V for 18 min. The membrane was blocked with PBS containing 3% skim milk at room temperature for 45 min and then washed five times with 0.1% Tween-20 in PBS. The following primary antibodies were added at 2 μg/mL: mouse anti-His (Invitrogen), rabbit polyclonal anti-MED25 (Abcam, Cambridge, MA, USA) and 2H2. After incubating at room temperature for 90 min with the primary antibody, the membrane washed again as above and incubated with the appropriate alkaline phosphatase labeled secondary antibody (Jackson ImmunoResearch, West Grove, PA, USA) at room temperature for 60 min. Following another wash step, the alkaline phosphatase substrate solution of nitroblue tetrazolium (NBT) and 5-bromo-4-chloro-3-indolylphosphate (BCIP) (Sigma Aldrich, Saint Louis, MO, USA) was added for incubation at room temperature for 3–5 min. Finally, the membrane was rinsed with distilled water.

### 2.8. Indirect Immunofluorescence Assay

Two days after transfection with the MED25 mammalian expression vector, the monolayer of adherent 293T cells on the plate was washed once with PBS and then fixed with PBS containing 4% paraformaldehyde (Sigma Aldrich) at room temperature for 10 min. Next, the cells were permeabilized using PBS containing 0.25% Triton X-100 at room temperature for 8 min and then blocked with PBS containing 5% Fetal bovine serum (FBS, Gibco, Waltham, MA, USA) at room temperature for 10 min. After washing once with PBS for 5 min, primary antibodies the same as the ones used for Western blot above were added separately at 60 μg/mL and incubated at 37 °C for 90 min. After washing three times with PBS, the fluorescent dye labeled secondary antibody Rhodamine Red-X Goat Anti-Rabbit IgG (H+L) and Alexa Fluor 488 Donkey Anti-Mouse IgG (H+L) (Invitrogen) were added for incubation at 37 °C for 40 min according to the user manual. Following another three washes, the cells were finally stained with the DAPI nucleic acid stain (Invitrogen) at room temperature for 5 min as the user manual described. After staining, the plate was washed once and sealed with 50% glycerol. Fluorescence signals were observed using the IX71 inverted research microscope (OLYMPUS) at 400× magnification.

### 2.9. ELISA

ELISAs were carried out as previously described [35]. Briefly, the capture reagent (2 μg/mL) was coated on 96-well microplates (CoStar, Tewksbury, MA, USA) at 4 °C overnight. The plates were then blocked with 3% bovine serum albumin (BSA) in PBS at 37 °C for 3 h. The antibody samples were serially diluted in duplicates, and then the bound antibody was detected by appropriate the HRP labeled secondary antibody (Jackson ImmunoResearch).

The competitive inhibition ELISA was performed as reported previously [36]. In brief, the microplates were coated with EV71 VLP, and blocking was performed as described above. 2H2 (5 μg/mL) was mixed with an equal volume of serially diluted MED25 protein (6.25–200 μg/mL) as the inhibitor or BSA as a control, and then the mixture was added to the plate. After washing, the plate-bound 2H2 was detected by peroxidase-conjugated AffiniPure Goat Anti-Mouse IgG (H+L) (Jackson ImmunoResearch).

In order to determine the endpoint titers, sera of immunized rabbits and EV71-infected patients, which were serially diluted, were reacted with antigen (VLP, OVA-P, OVA or MED25) coated on plates. The secondary antibodies Peroxidase-AffiniPure Donkey Anti-Human IgG (H+L) and Perox-AffiniPure Donkey Anti-Rabbit IgG (H+L) (Jackson ImmunoResearch) were used to detect the plate-bound antibodies according to user manual. The endpoint titer was defined as the highest dilution with an OD value higher than the cut-off value (2.1-fold higher than the negative control signal).

### 2.10. Determination of Affinity of 2H2 to MED25

The affinity of 2H2 to MED5 was determined by the Octet Red96 system (ForteBio, Menlo Park, CA, USA). The 2H2 mAb was biotinylated using the ImmunoProbe™ Biotinylation Kit (Sigma Aldrich) and then loaded at a concentration of 50 μg/mL onto the streptavidin sensors (ForteBio), which were equilibrated in PBS. Next, the sensors were transferred into the MED25 protein solution (MED25 dissolved in PBS) at the 2-fold serial dilutions concentration of 18.75–300 μg/mL, followed by PBS to dissociate nonspecifically bound components. Dissociation constants were calculated from raw data by the analysis software accompanying the Octet Red96 system (version 6.3, ForteBio).

### 2.11. Immunohistochemistry

Explanted mouse (healthy Balb/c mice, 2 weeks old) brain stem slides were incubated with 2H2, rabbit anti-VLP serum or EV71-infected patient serum at dilutions of 1/150 (5 μg/mL), 1/100 and 1/100 for 20 min. Because of the homology of brain stem tissue and the 2H2 mAb, 2H2 was labeled with HRP using glutardialdehyde as described previously [37]. After washing with PB (PBS without salt), the anti-rabbit or anti-human secondary antibody (Jackson ImmunoResearch) was added and incubated at room temperature for 15 min, followed by washing as above. Finally, immunoreactivity was detected as the brown color generated by adding diaminobenzidine (DAB) (Invitrogen) as described by the manufacturer. Images of the immunohistochemical stains were taken at 200× magnification.

### 2.12. Live Small Animal *in Vivo* Imaging

The mice (2-week-old Balb/c) were randomly divided into six groups of five mice each (Figure 8). First, 2H2 was labeled with the infrared dye Alexa Fluor 750 using the SAIVI^TM^ rapid antibody labeling kit (Invitrogen) according to instructions in the manual. 2H2-infrared (125 μg/mouse) was injected into the caudal vein of mice that had been inoculated with EV71 virus (C4a strain, 10^8^ TCID_50_/mL, 300 μL/mouse) for 0, 3 or 7 days. At 3 h after injection of 2H2-infrared, the mice manifestations were recorded and the distribution of the dye was visualized using the KODAK *In-Vivo* Multispectral System FX (KODAK) with the 775 nm channel. Meanwhile, the isotype control for 2H2 was processed and detected in parallel.

## 3. Results

### 3.1. Alignment of EV71 Proteome Fragments to MED25 of Various Species

**Figure 1:**
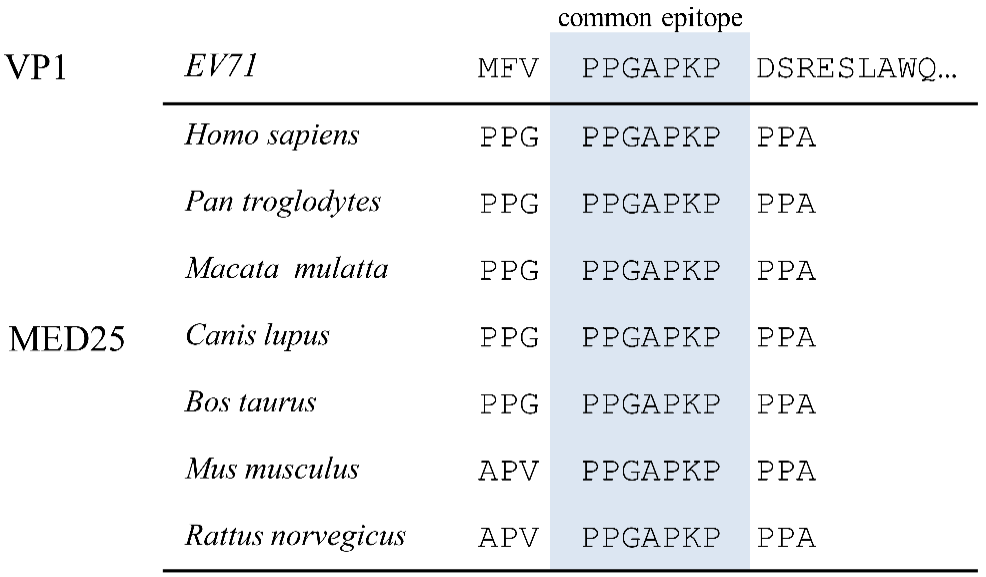
Alignment of amino acid sequences of common epitope in enterovirus 71 (EV71) virus protein 1 (VP1) with mediator complex subunit 25 (MED25) of various species. Shown are the common epitope sequence (highlighted) and flanking amino acids.

In order to identify common epitope(s) between viral and human antigens, the amino acid (aa) sequence of the EV71 proteome was divided into 10-aa fragments and then used to conduct BLAST searches in the GenBank human proteome database. The search identified the protein MED25, which is linked to human diseases including neurological disorders [38], with a 7-aa sequence (PPGAPKP, which is conserved in EV71, see supplementary file Figure S2) match with EV71 VP1. Here, we are only interested in the common epitope, so there were no more data on it provided. The alignment of this VP1 fragment with MED25 of various species including humans suggests that this common epitope is highly conserved in mammalian species (Figure 1).

### 3.2. Localization of Common Epitope on Canyon Slope Surface of Viral Capsid Exterior

**Figure 2:**
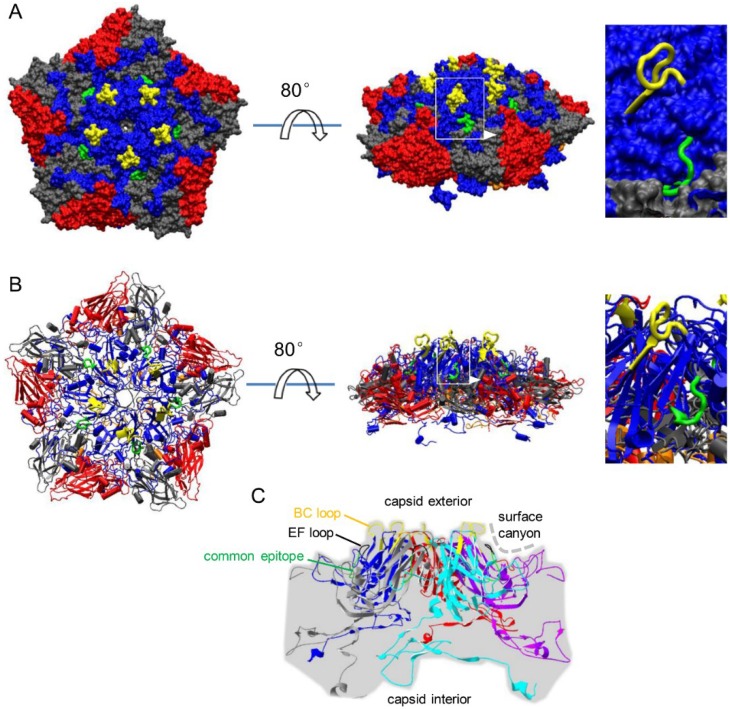
Location of common epitope on the viral capsid surface. (**A**) A pentamer (VP1, blue; VP2, red; VP3, gray; VP4, orange) of the EV71 viral capsid (pdb ID: 3VBS) is shown as an electrostatic surface model, where the common epitope is highlighted in green, and the BC loop is highlighted in yellow; (**B**) the cartoon of the pentamer of which color scheme is identical to that in panel A; and (**C**) the location of common epitope is indicated and localized with BC loop and EF loop in the pentamer of VP1. Colors of these five VP1 copies were assigned randomly.

The crystal structure of human EV71 (pdb ID, 3VBS) was modified to define the location of the common epitope on the capsid surface. The viral pentamer (VP1, VP2, VP3 and VP4) was rendered as an electrostatic surface model (Figure 2A) and a cartoon (Figure 2B). From these two diagrams, we firmly concluded that the common epitope is located on the sloped surface of the canyon on the capsid exterior surface and closely beneath the EF loop (Figure 2C). The BC loop, which is at the peak of the pentamer on the outer viral capsid, and the EF loop are indicated in the illustrations in order to place the common epitope into spatial context. The location of the common epitope on the MED25 molecule surface cannot be shown, because there is still no crystal structure of full-length MED25 reported hitherto.

### 3.3. Preparation of mAb against the Common Epitope and the Reactivity of it with MED25

**Figure 3:**
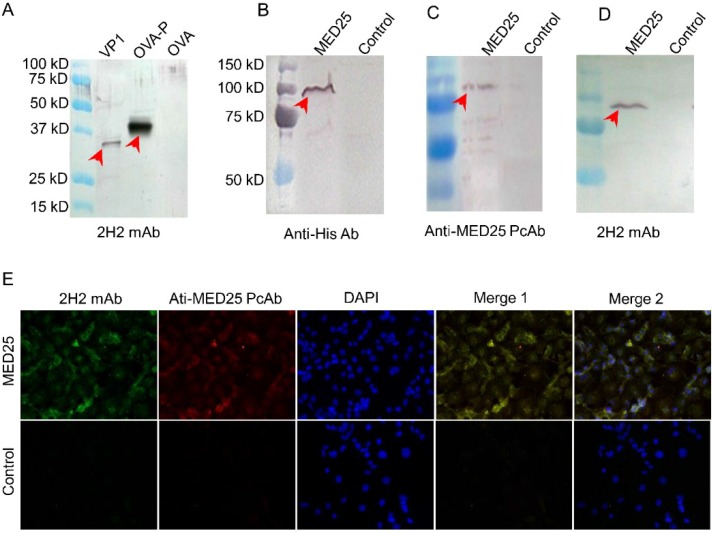
Reactivity of 2H2 to MED25 and common epitope. (**A**) Cross-reactivity of the 2H2 mAb against the common epitope. 2H2 mAb was generated using the common peptide (PPGAPKP) coupled on OVA. The cross-reactivity was detected by Western blot. 1 μg VP1, 5 μg OVA-P and 5 μg OVA were loaded on the gel. 2H2 mAb was used at a final concentration of 2 μg/mL. Specific bands are indicated by the red arrows. OVA, vector; OVA-P, vector with the common epitope peptide; (**B**) The lysate of 293T cells transfected with MED25 expression vector was stained by anti-His, cells were transfected with the empty expression vector as a control. MED25 was probed by these antibodies in an independent Western blot experiment, and 10^5^ cells lysate was loaded in each cell on gel. Specific band is indicated by the red arrow; (**C**) The lysate was stained by anti-MED25 commercial polyclonal antibody, and the other conditions were the same as panel B; (**D**) The lysate was stained by 2H2, and the other conditions were the same as panel B; (**E**) 293T cells transfected with MED25 expression plasmid and empty plasmid (control), respectively, were indirectly stained with 2H2 (FITC, green) and anti-MED25 (rhodamine, red) antibodies. The nuclei were stained with DAPI (blue). Merge 1: green + red. Merge 2: green + red + blue. The isotype antibody was included as the control to monitor specific staining. Images were obtained at a magnification of 400×.

Using the hybridoma technique, we prepared the 2H2 mAb against the common epitope with the aim of studying its cross-reactivity to MED25 and EV71 VP1. The reactivity of the mAb to EV71 virus-like particle (VLP) or common epitope linked to ovalbumin (OVA) was determined by Western blot. Positively stained bands appeared in the lane loaded with the VLP or OVA-common epitope (OVA-P), while the OVA control lane was negative (Figure 3A). These results suggested that the specific mAb against the common epitope (PPGAPKP) was prepared successfully and could be used in the following experiments. We also determined that the 2H2 mAb did not neutralize the entry of all genotypes of EV71 viruses into 293S cells (293 cells with stable expression of SCARB2 established in our laboratory [39]).

Next, the reactivity of 2H2 to MED25 protein expressed in 293T cells was evaluated. The Western blot analysis indicated that MED25 was successfully expressed, as it was detected as a specific band when stained by an anti-His tag antibody, while the control was negative (Figure 3B). To further confirm the successful expression of the MED25 protein, a commercial anti-MED25 polyclonal antibody was used, and it produced a band of a size corresponding to that detected by the anti-His tag antibody (Figure 3C). Finally, the reactivity of 2H2 to MED25 was verified by Western blot using the lysate of cells expressing MED25 as the antigen, and an identical band to those above was specifically stained (Figure 3D). However, the control was absolutely negative, suggesting the expression level of endogenous MED25 protein was too low to be detected by Western blot. These results demonstrated that the anti-common epitope 2H2 mAb was confirmed to react with both VP1 and MED25.

Additionally, we detected binding of 2H2 to 293T cells expressing MED25 using immunofluorescence. The 2H2 mAb labeled the perinuclear region of 293T cells (Figure 3E) and colocalized with an anti-MED25 specific antibody (Figure 3E), suggesting that MED25 is the common antigen of 2H2 in 293T cells. And the control was negative, the most possible reason is that the expression level of it in 293T cells was too low.

### 3.4. MED25 and VP1 Contain the Common Antigen Recognized by 2H2 mAb

**Figure 4:**
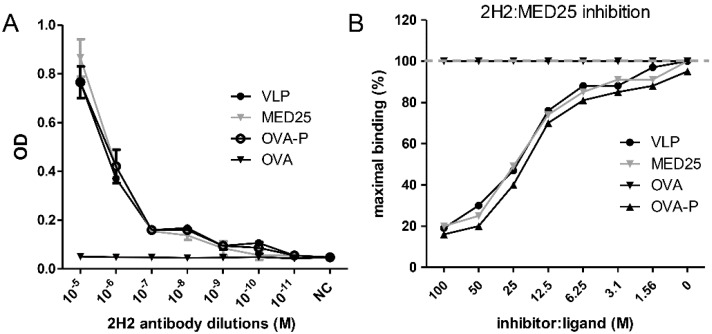
VLP and MED25 are co-antigens of 2H2. (**A**) Binding activity of 2H2 to VLP and MED25 were evaluated by ELISA as described in Materials and methods. OVA-P and OVA were used as positive and negative control respectively; (**B**) Inhibition of 2H2 binding to immobilized EV71 VLP was evaluated by ELISA as described in Materials and methods. VLP and OVA-P were used as the positive control and OVA as the negative control. The y-axis represents OD as the percentage of maximal binding, which is determined as the mean reading without inhibitors (100% binding is marked by the gray dot line for comparison). The x-axis indicates the molar ratio of inhibitor to plate-immobilized ligand.

2H2 exhibited similar affinities to MED25 and VP1 under common ELISA conditions, with no significant difference (*p* < 0.0001) in OD values at the same antibody dilution when reacting with the two antigens (Figure 4A). The equivalent affinities of 2H2 to MED25 and VLP (VP1) were also demonstrated by an inhibition ELISA. MED25 (inhibitor) completely abrogated 2H2 (ligand) binding to VLP at a high ratio, which was similar with the inhibition of 2H2 binding to VLP by VLP itself (Figure 4B).

### 3.5. Determination of Binding Affinity of 2H2 to MED25

The binding affinity of 2H2 was determined using serially diluted MED25 (18.5–300 μg/mL). After analysis of the raw data, the Kd value of 8.01 × 10^−7^ M (the normal range is 10^−9^–10^−6^ M) was obtained, representing a better-than-average binding affinity.

### 3.6. Detection of 2H2-Like Antibodies in Rabbit anti-VLP and anti-MED25 Sera and in EV71-Infected Patient Serum

**Figure 5:**
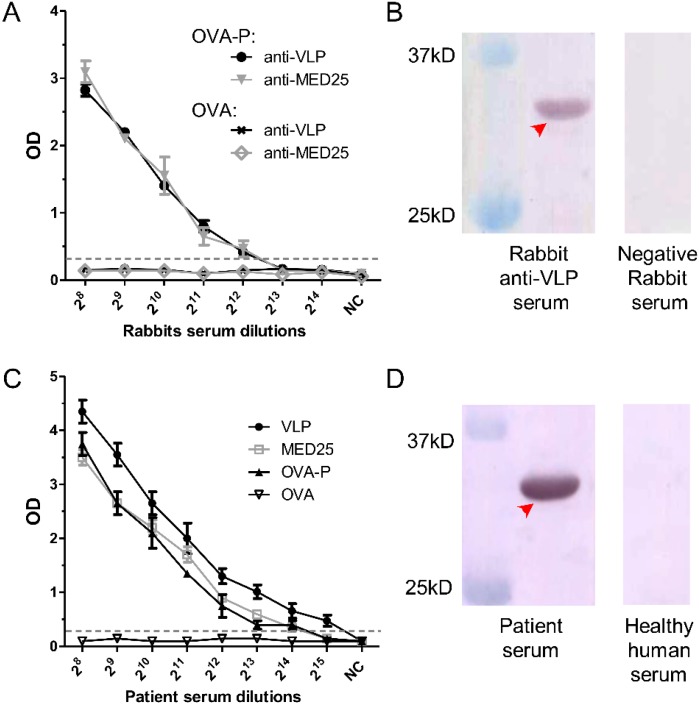
Presence of 2H2-like antibodies against the common epitope in rabbit immune sera and patient serum. (**A**) Titration of serum from rabbits immunized with VLP or MED25 using OVA-P as the capture ligand and OVA as the negative control. The cut-off value is indicated by the gray dotted line; (**B**) The presence of 2H2-like antibodies in rabbit anti-VLP serum was confirmed by Western blot with the recombinant MED25 as the capture antigen. Negative rabbit serum was used as negative control. The specific band is indicated by the red arrow; (**C**) Titration of 2H2-like antibodies in patient serum using the capture VLP, MED25 or OVA-P as the capture ligand and OVA as the negative control. The gray dotted line indicates the cut-off value line defined as 2.1-fold greater than the average of the negative control value; (**D**) The presence of 2H2-like antibodies in patient serum was confirmed by Western blot with the recombinant MED25 as the capture antigen, and healthy human serum was used as negative control. The specific band was indicated by the red arrow.

To determine whether 2H2-like antibodies are generated in animals immunized with MED25 or EV71 VLP, rabbit anti-MED25 and anti-VLP sera were used to bind OVA-P in an ELISA. 2H2-like antibodies in the anti-VLP serum were detected, with the highest positive dilution of 2^12^, and the same result was observed with the anti-MED25 serum; meanwhile, the OVA control was negative (Figure 5A). In order to clarify whether these 2H2-like antibodies also exist in humans, serum from an EV71-infected patient was used to bind VLP, MED25 and OVA-P in an ELISA with OVA as a negative control. The titers to these antigens in the patient serum reached up to 2^14^ for MED25 and OVA-P and to 2^15^ for VLP (Figure 5C). We then confirmed the presence of these 2H2-like antibodies in the EV71-infected patient and rabbit anti-VLP serum by binding to recombinant MED25 in a Western blot, and specific bands were observed in the appropriate location (Figure 5B,D), however, the negative rabbit serum and healthy human serum did not stain the antigen.

### 3.7. Dynamics of 2H2-Like Antibody Generation in Rabbit Immune Serum and Endpoint Titer in Patient Serum

**Figure 6:**
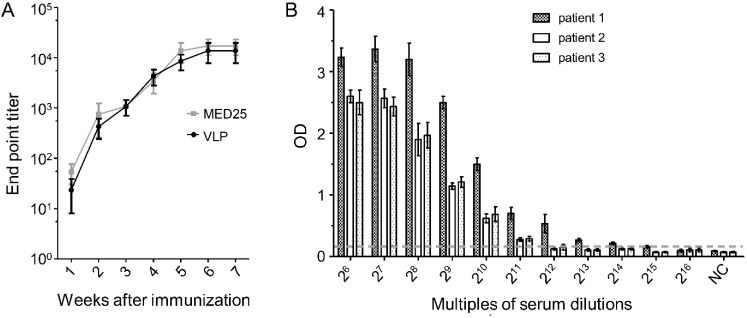
Kinetics of 2H2-like antibodies in immunized animals and prevalence of 2H2-like antibodies in patient serum. (**A**) Rabbits were immunized with MED25 or VLP, and serum samples collected each week were titrated by ELISA using OVA-P as the capture ligand; (**B**) Endpoint titers of 2H2-like antibodies in sera of randomly selected EV71-infected patients were determined by ELISA using OVA-P as the capture ligand. The cut-off value (2.1-fold of negative control value) is indicated by the dotted line.

The dynamic generation of 2H2-like antibodies in the serum of VLP and MED25 immunized rabbits was examined using OVP-P in an ELISA. 2H2-like antibodies were detected at one week after immunization and increased over time, with the highest titer observed at the seventh week (Figure 6A). In order detect the prevalence of 2H2-like antibodies in EV71-infected patient sera, we screened 30 clinical samples selected randomly from a panel of 200 samples, and all of them were positive. Subsequently, we randomly chose 3 of these 30 positive samples and determined their endpoint titers to be 2^14^ (patient 1), 2^11^ (patient 2) and 2^12^ (patient 3) (Figure 6B).

### 3.8. Cross-Reactivity of 2H2, Rabbit anti-VLP Serum and EV71-Infected Patient Serum in Brain Tissue

**Figure 7:**
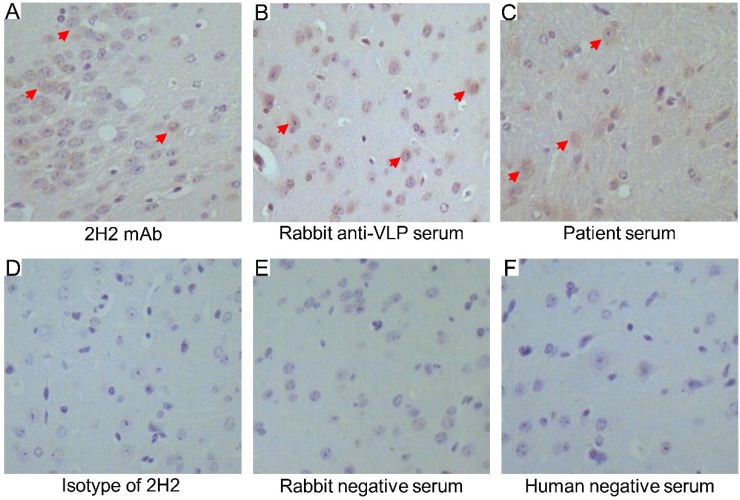
Cross-reactivity of 2H2, EV71 VLP-immunized rabbit serum and EV71-infected patient serum with mouse brain stem tissue. (**A**) The tissue slide was stained with 2H2 directly labeled with HRP, positive specific stains are indicated by red arrows; (**B**) tissue slides were stained indirectly with anti-VLP serum from rabbits, positive specific stains are indicated by red arrows; (**C**) tissue slides were stained indirectly with EV71-infected patient serum, positive specific stains are indicated by red arrows; (**D**) the HRP-labeled isotype-matched antibody was used as the negative control for 2H2 mAb; (**E**) the rabbit negative serum was used as the negative control for rabbit anti-VLP serum; and (**F**) the human negative serum from healthy human was used as the negative control for EV71-infected patient serum. Images were obtained at a magnification of 200×.

We next evaluated the cross-reactivity of 2H2, anti-VLP serum and EV71-infected patient serum by immunohistochemistry using tissue from the healthy mouse brain stem. Positive reactivity was indicated by the brown stain on the brain tissue section stained with 2H2, anti-VLP serum and patient serum (Figure 7A–C, respectively). By contrast, the isotype of 2H2 and negative serum as controls did not stain the tissue sections (Figure 7D–F). Additionally, we also confirmed the expression of MED25 in brain tissue (see Supplementary File Figure S3).

### 3.9. Reactivity of 2H2 to Brain Tissue and the Distribution *in Vivo*

**Figure 8:**
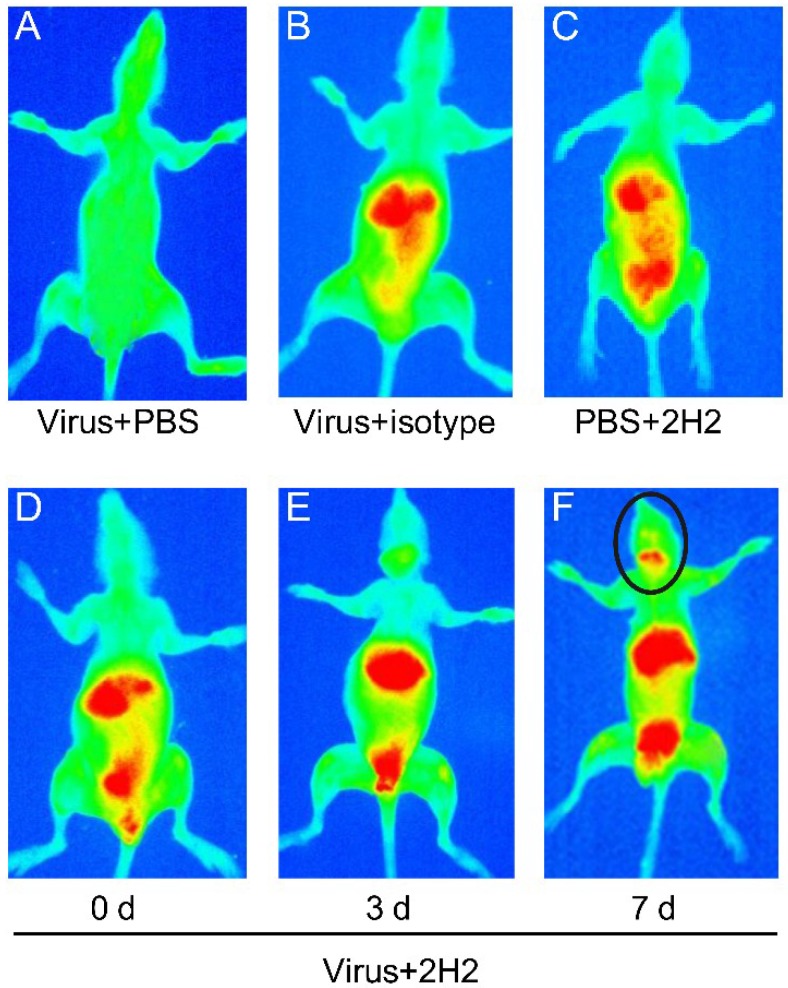
Reactivity of 2H2 with MED25 and the distribution *in vivo*. (**A**) PBS was injected after inoculation of virus served as blank for the infrared dye-labeled 2H2; (**B**) the substitution of isotype-matched antibody for the infrared dye-labeled 2H2 served as negative control; (**C**) meanwhile, PBS was substituted for virus as the control and processed parallel to the virus-inoculated group (0, 3 and 7 d); (**D**–**F**) Mice were injected with 2H2-infrared at 0, 3 and 7 d after virus inoculation. The red color represents the distribution of 2H2 *in vivo*, and the black circle marks the distribution of 2H2 in the brain stem region.

Next, we examined the reactivity of 2H2 to brain tissue by using live small animal *in vivo* imaging. 2H2 was labeled with a near infrared dye and then injected into the mouse tail vein at 0, 3 and 7 days after inoculation of EV71 virus. The same results were obtained from five mice in each experiment group. The 2H2 was mainly distributed in the liver after injection initially, but then it transferred to the kidney and bladder over time. As expected, 2H2 was transferred to the brain stem region of mice at seven days after inoculation with the virus. The mouse injected with virus and phosphate buffer solution (PBS) as the blank control did not display any signal (red color) (Figure 8A). No signal in the brain or brain stem was detected in the mouse injected with virus and an isotype-matched control antibody (Figure 8B), nor in the brain region of the no-virus control mouse injected with PBS and 2H2 (Figure 8C). Injecting virus at 0 and 3 days before injecting the 2H2 antibody did not induce the transfer of the antibody to the brain stem (Figure 8D,E). However, in mice infected with EV71 for seven days, 2H2 was transferred and accumulated in the brain stem region (Figure 8F). At the same time, these mice showed signs of depression but not classical neurological symptoms.

## 4. Discussion

The severe neurological complications of HFMD pose a major threat to infant health, which have not yet been avoided by vaccination or eliminated by drug treatment. Understanding the pathogenesis of this disease would be very useful in developing therapies to control it. However, the neurological effects of EV71 are still not completely clear despite much research on this viral agent of HFMD. It is widely known that autoimmune responses triggered by an autoantibody binding to the autoantigen activate immune cells such as macrophages (microglia and astrocytes in the nervous system) to secrete cytokines (e.g., IL-6, IL-10, IL-13, TNF-α and IFN-γ) that contribute to the development of neurological complications [22,23,24,25,26,27,28]. Many previous studies have reported on autoimmune disease induced by virus infection (for instance, ankylosing spondylitis, Guillain-Barre syndrome and primary biliary cirrhosis) [40,41,42], as well as the effects of autoreactive antibodies on encephalitis [43,44,45]. Therefore, autoimmune reactive antibodies cannot be excluded as mediators of EV71-associated neurological disease.

In this study, we identified through peptide scanning a common epitope contained within the EV71 VP1 and human MED25 proteins, which were both recognized by high titered antibodies induced by EV71 infection in patients. The 2H2 mAb against the identified common epitope was prepared, and its reactivities to EV71 VP1 and VLP as well as to MED25 were evaluated. The autoantibody 2H2 showed an affinity to VP1 and MED25 under both non-native (Western blot) and native (ELISA) conditions. Additionally, the putative autoantigen MED25 identified in this study is expressed ubiquitously with highest levels in the spleen, heart, brain stem, kidney, placenta and skeletal muscle [30]. However, because the physiological function of brain is sensitive to tissue damage, the manifestations caused by autoimmunity can be most apparent in this organ. So, our findings may be an important step in understanding the pathogenesis of neurological complications associated with EV71 infection.

As results shown, no significant difference between the affinities of 2H2 to VLP and MED25 was observed (Figure 4A), and MED25 inhibited the binding of 2H2 to VLP at a level similar to the inhibition of 2H2 in binding to VLP by VLP itself (Figure 4B). This result suggested that 2H2-like antibodies in patient serum would bind to both the virus and the autoantigen. Additionally, we determined the affinity of 2H2 binding to MED25 using the ForteBio Octet System, which applies the optical interference theory and produces highly accurate and reproducible data. Serial five-fold concentrations of MED25 were analyzed, and each of the concentrations yielded the same results (Kd = 8.01 × 10^−7^ M), further confirming the affinity of 2H2 to MED25. From these results, we easily concluded that 2H2 can bind to MED25 *in vitro*. The 2H2 mAb also showed no neutralizing activity, suggesting that the high 2H2-like antibody levels detected in patient serum may favor disease development by generating an autoimmune response while allowing for the persistence of viral replication.

Another aim of this study was to verify the potential association of autoantibodies with neurological implications of EV71 infection. We first tested serum from rabbits immunized with VLP or MED25 and found that both of them could induce high levels 2H2-like antibodies in the animal immune serum (Figure 5A). Next, we analyzed the EV71-infected patient serum, and the endpoint titer was two times higher than that of the rabbit immune serum (Figure 5A,B). Additionally, the reactivity was confirmed by Western blot. Meanwhile, the kinetics of 2H2-like antibody induction by VLP was determined and the wide prevalence of 2H2-like antibodies in a panel of patient serum samples was also confirmed. These findings above suggested that high levels of 2H2-like autoreactive antibodies were generated in immunized experimental animals and individuals infected with EV71 virus, which supported the possibility of virus-mediated autoimmune responses and would be a primary factor leading to autoimmune disease.

Immunohistochemical analysis showed that not only the 2H2 mAb but also the 2H2-like antibodies induced by VLP immunization or EV71 infection specifically bound to the mouse brain stem tissue sections. This result indicated that the 2H2 and 2H2-like antibodies could react to brain tissue *in vitro*. Due to the conservation of MED25 proteins among mammalian species, this result may be extended to humans. A similar positive immunohistochemical reactivity using a human specimen was reported previously [29].

In order to visualize the distribution of 2H2 *in vivo*, a live small animal imaging experiment was performed. Because the antibody could not be transferred through an intact blood brain barrier (BBB) to the central nervous system, untreated control mice exhibited negative results. A previous study showed that the BBB permeability could be increased by EV71 infection [29]. Based on this finding, mice in the live imaging experiment were infected with EV71 virus (C4a strain) prior to administration of the infrared dye-labeled 2H2 mAb (2H2-infrared). At secen days after infection, 2H2-infrared was distributed in the brain stem region. However, 2H2-infrared was not detected in the brain stem at day 0 and 3 post-infection, most likely because the virus infection had not been established to sufficiently increase the BBB permeability to allow for the antibody to cross into brain tissue. Although 2H2-infrared was distributed in the brain stem, the mice did not exhibit classical neurological symptoms, such as hind limp paralysis, and only showed signs of depression. Several reasons may account for this phenomenon. The virus-induced neurological disease is highly complex, and the autoimmune response may not be the only cause. In previous studies, EV71 was found to only infect suckling mice [46,47] up to two weeks of age [48]. However, in our study the mice were three weeks old when injected with 2H2-infrared. Additionally, the common epitope has been detected in VP1 of coxsackievirus A16 (CA16), which is a secondary pathogen associated with neurological complications and always co-infected with EV71 [49,50]. However, we did not combine the CA16 with EV71 in this study to investigate the cause of neurological disease. Therefore, further study is needed to verify the potential immunopathogenesis of co-infection of EV71 and CA16.

## 5. Conclusions

In summary, we identified for the first time a common epitope (PPGAPKP) of human MED25 and EV71 VP1. The mAb against this epitope was shown to bind to VP1 and MED25 at a higher-than-average level of affinity not only *in vitro* but also *in vivo.* Meanwhile, both EV71 VLP and the MED25 could induce high levels of antibodies against the common epitope after immunization of experimental animals. Additionally, high levels autoreactive antibodies were found in sera from EV71-infected patients. All of these findings implicated the autoimmune response as a pathogenic mechanism of EV71-associated neurological disease. This study provides a theoretical foundation for further studies and considerations in vaccine design for HMFD.

## Supplementary Files

Supplementary File 1
