# Supplementary material for: Identification of a Common Epitope between Enterovirus 71 and Human MED25 Proteins Which May Explain Virus-Associated Neurological Disease
Source: Viruses. 2015 Mar 27;7(4):1558–77. doi: 10.3390/v7041558 (PMC4411665; doi:10.3390/v7041558)
Supplement: Supplementary File 1 [file viruses-07-01558-s001.pdf]

Supplementary Materials

Expression of Truncated Recombinant MED25 in *E. coli*

In order to obtain a large amount of recombinant MED25, the truncated protein which retained the common epitope at the C-terminus was expressed by transformation of *E. coli*. The MED25 gene fragment (241–1080 bp) with a His tag sequence at the 3'-terminal end was inserted into the pET-28a(+) plasmid (Novagen), which was then transformed into BL21(DE3) Chemically Competent *E. coli* (Invitrogen) based on the procedure described in the manual. The transformed *E. coli* was incubated at 37 °C by shake cultivation at the speed of 220 rpm until the OD reached 0.8 (about 150 min), and then isopropy-β-D-thiogalactoside (IPTG) was added to the final concentration of 1 mM to induce protein expression for a further 10 h of shake cultivation. Subsequently, the MED25 fragment was purified by nickel affinity chromatography using the ÄKTA purification system (GE Healthcare) according to the manufacturer's instructions.

By SDS-PAGE analysis, the MED25 protein fragment was observed in the lysate of *E. coli* transformed with the expression plasmid but not the control (Figure S1). After purification, the fragment was identified by western blot, and a specific band was detected by 2H2, anti-His and anti-MED25 antibodies (Figure 4B–D, respectively).

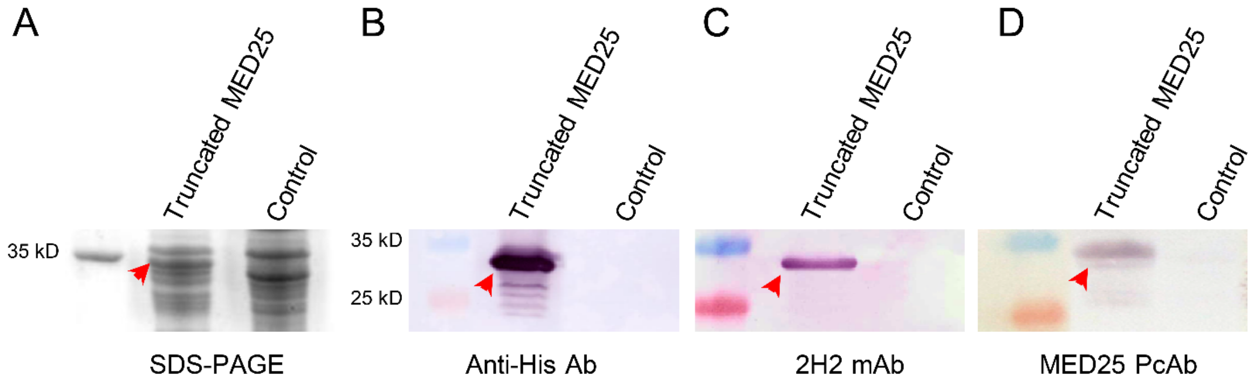

Figure S1. Expression and detection of truncated MED25 protein. (**A**) Lysates (generated from 100 µL of inocula with the OD value was 1.2) of *E. coli* expressing MED25 and control *E. coli* transformed with empty plasmid were analyzed by SDS-PAGE. The target band is indicated by the red arrow; (**B**) The truncated MED25 was detected using an anti-His mAb by western blot. The specific band is indicated by the red arrow; (**C**) The truncated MED25 was detected using 2H2 mAb by western blot. The specific band is indicated by the red arrow; (**D**) The truncated MED25 was detected using a commercial anti-MED25 polyclonal antibody by western blot. The specific band is indicated by the red arrow.

Group	Alignment (positions 188–200; 190, 200 marked)	Serotypes	GenBanks	Strains/Isotates	Countries	Years
Majority	M Y V P P G A P K P D S R					
HEV-A	. F . . . . G . .	EV71(A)	ACS12928	1906-Luan(CHN)-08	China	2008
	. F	EV71	AEI71312	JP52/Sm/W/10	Japan	2010
	. F . . . . K . .	EV71(B4)	AEM23777	02205	Thailand	2006
	. F	EV71(C2)	AFJ15580	C2/EV71/80/PHL/2005	Japan	2005
	. F	EV71(C4)	AFL71292	Cixi.CHN/016/2011	China	2011
	. F	EV71	AHG54563	SK091/2013	Malaysia	2013
	. F	EV71	AIL54930	13390/SD/CHN	China	2013
	. F	EV71	AIW00794	163-Henan-2014	China	2014
	. F	EV71	BAO93836	933-Yamagata-2013	Japan	2013
	. F	EV71	BAP27872	EV71/25-1034/osaka.JPN/2013	Japan	2014
	. . . . D G .	CAV2	BAD36910	CA2/80250/Hiroshima.JP/04	Japan	2004
	. . . . D G .	CAV2	AJK93829	JB141330351-CA2	China	2013
	. . . . D A .	CAV4	ACT52614	98401/SD/CHN/1998/CA4	China	1998
	. . . . D A .	CAV4	BAH24182	JR	Japan	2008
	. . . . D A .	CAV4	AGR84760	JB141230147	China	2012
	. . . . D . .	CAV6	AFN66602	10032/SD/CHN/2010/CA6	China	2010
	. . . . D . .	CAV6	AHG54568	SK018/2013	Malaysia	2013
	. . . . D . .	CAV6	BAK54005	shizuoka_1	Japan	2010
	. . . . V . . G .	CAV10	ACS88972	H587F/SD/CHN/2008/CA10	China	2008
	. . . . G .	CAV10	AHF49571	SJZ10-1514T/HeB/CHN/2010	China	2010
	. . . . G .	CAV10	BAC92728	CA10/20096/Hiroshima.JP/03	Japan	2003
	. . . .	CAV16	CAL23420	UM17115/MAL/00	Malaysia	2000
	. . . .	CAV16	ADD84741	Siriraj06/TH/05	Thailand	2005
	. . . .	CAV16	AEM23782	00332	China	2005
	. . . .	CAV16	AFL91468	PM-1824818-07	Malaysia	2007
	. . . .	CAV16	AIW00882	25-Henan-2014	China	2014
	. . . .	CAV16	BAK26678	2441-Yamagata-2005	Japan	2005
	. . . .	CAV16	BAO79777	110258/CA16/kobe/2011	Japan	2011
	. . . .	CAV16	CAL23413	TS1-2000/THAI/00	Thailand	2000
HEV-B	. . . . . . G . I . A K V	CAV9	BAD12599	Fukuoka City03/171	Japan	2004
	. . . . . . G . I . A K V	CAV9	ACT98442	04318/SD/CHN/2004/CA9	China	2004
	. . . . . . G . V . . K V	CBV3	ACT98478	37010408199/SD/CHN/2008/CB3	China	2008
	. . . . . . G . V . . K V	CBV3	AFV34692	M475	India	2009
	. . . . . . G . V . . K V	CBV3	BAQ00093	Se6/Fukushima/JPN/2013	Japan	2013
	. . . . . . G . V . . K V	CBV5	AHK27233	SWS/CHN/AM/07/CB5	China	2010
	. . . . . . G . V . . K V	CBV5	BAD12610	Fukuoka City03-158	Japan	2003
	. . . . . . G V V . A . V	EV69	AEX15068	N-970	India	2011
HEV-C	. . . . R . S K W	EV95	AGF90648	95_T08-234	Chad	2008
	. . . . V . Q Q W	EV-C	ABN79676	12-04-856	Congo	2006
	. . I . . . . . Q . T A W	CAV24	ACT98437	99053/SD/CHN/1999/CA24	China	1999
	. . . . V . G K W	PV1	CAB65072	PV1/6402/ISR87	Israel	2000
HEV-D	. F . . T . . L T . E K Q	EV68	AGO02239	ITA/34800/10	Italy	2010
	. F . . T . . L T . E K Q	EV68	AGR88908	CQ5914	China	2012
	. F . . T . . L T . E K Q	EV68	AHV84986	HEV196011	Kenya	2011
	. F . . T . . L T . E K Q	EV68	BAP76278	TTa-11-Ph344_VP1	Philippines	2011

Figure S2. Alignment of amino acid sequence of common epitope in human enterovirus (HEV) VP1 with different serotypes. Shown are the common epitope sequence (red box) and flanking amino acids.

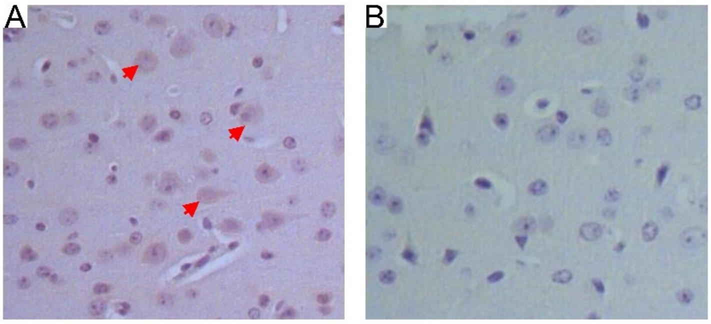

Figure S3. Confirmation of the expression of MED25 in mouse brain stem tissue. The tissue slide was stained with commercial anti-MED25 antibody (A), and the HRP-labeled isotype-matched antibody was used as the negative control (B). Positive stains are indicated by red arrows. Images were obtained at a magnification of 200×.
